# Supplementary material for: Causal Relationship between Aging and Anorexia Nervosa: A White-Matter-Microstructure-Mediated Mendelian Randomization Analysis
Source: Biomedicines. 2024 Aug 16;12(8):1874. doi: 10.3390/biomedicines12081874 (PMC11351342; doi:10.3390/biomedicines12081874)
Supplement: Supplementary file 1 [file biomedicines-12-01874-s001.zip › Supplementary Figure/Figure S9/Leaveoneout plot of MR analysis for ubm-b-1626 to AN.pdf]

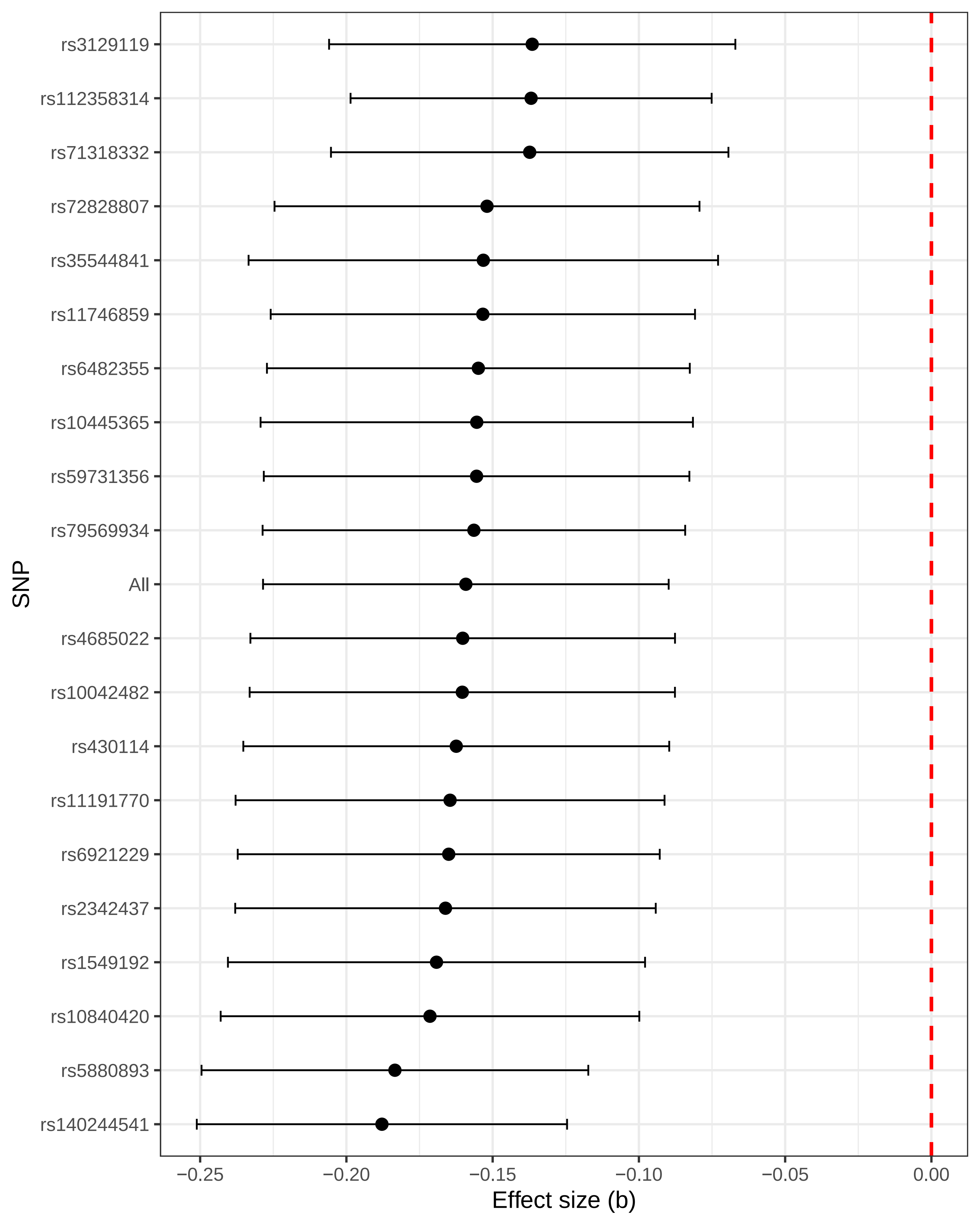
rs3129119
rs112358314
rs71318332
rs72828807
rs35544841
rs11746859
rs6482355
rs10445365
rs59731356
rs79569934
All
rs4685022
rs10042482
rs430114
rs11191770
rs6921229
rs2342437
rs1549192
rs10840420
rs5880893
rs140244541
SNP
−0.25
−0.20
−0.15
−0.10
−0.05
0.00
Effect size (b)
